# Peters Anomaly in a Miniature Schnauzer Documented by Spectral‐Domain Optical Coherence Tomography: A Case Report

**DOI:** 10.1002/ccr3.72136

**Published:** 2026-02-22

**Authors:** Yukihiro Miwa, Deokho Lee, Yuri Inukai

**Affiliations:** ^1^ Aichi Animal Eye Clinic Aichi Japan; ^2^ Laboratory of Photobiology, Keio University School of Medicine Tokyo Japan; ^3^ Korean Institute of Nutrition, Hallym University Chuncheon Republic of Korea

**Keywords:** canine, genetics, miniature schnauzer, peters anomaly, spectral‐domain optical coherence tomography

## Abstract

A 10‐month‐old intact male Miniature Schnauzer was presented for evaluation of a unilateral corneal opacity in the right eye. Ophthalmic examination revealed a broad paraxial corneal opacity involving the posterior stroma to the Descemet's membrane, with multiple strands of tissue extending from the iris collarette to the posterior cornea. Spectral‐domain optical coherence tomography (SD‐OCT) revealed focal absence of the Descemet's membrane and a defect in the corneal endothelium in the affected area. Based on the clinical and imaging findings, the case was diagnosed as Peters anomaly. To the authors' knowledge, this is the first report of Peters anomaly in a dog documented by SD‐OCT.

## Key Clinical Message

This case highlights Peters anomaly in a dog, documented by spectral‐domain OCT, demonstrating focal absence of Descemet's membrane and iridocorneal adhesions. OCT provides in vivo confirmation of congenital anterior segment dysgenesis and is a valuable non‐invasive tool for veterinary diagnosis and education.

## 1 Introduction

Peters anomaly is a rare congenital malformation of the anterior segment, characterized by central corneal opacity and adhesions between the iris and posterior cornea [1]. This anomaly results from defective separation of the lens vesicle from the surface ectoderm during embryogenesis, leading to absence or hypoplasia of the Descemet's membrane and corneal endothelium in the affected area [2].

Although Peters anomaly has been reported in humans [3], cats [4], and dogs [5], veterinary literature contains few canine cases. In this study, we describe a case of Peters anomaly in a Miniature Schnauzer in which spectral‐domain optical coherence tomography (SD‐OCT) enabled in vivo visualization of focal Descemet's membrane and corneal endothelial defects that have previously been described only in histopathological studies.

## 2 Case History/Examination

**FIGURE 1:**
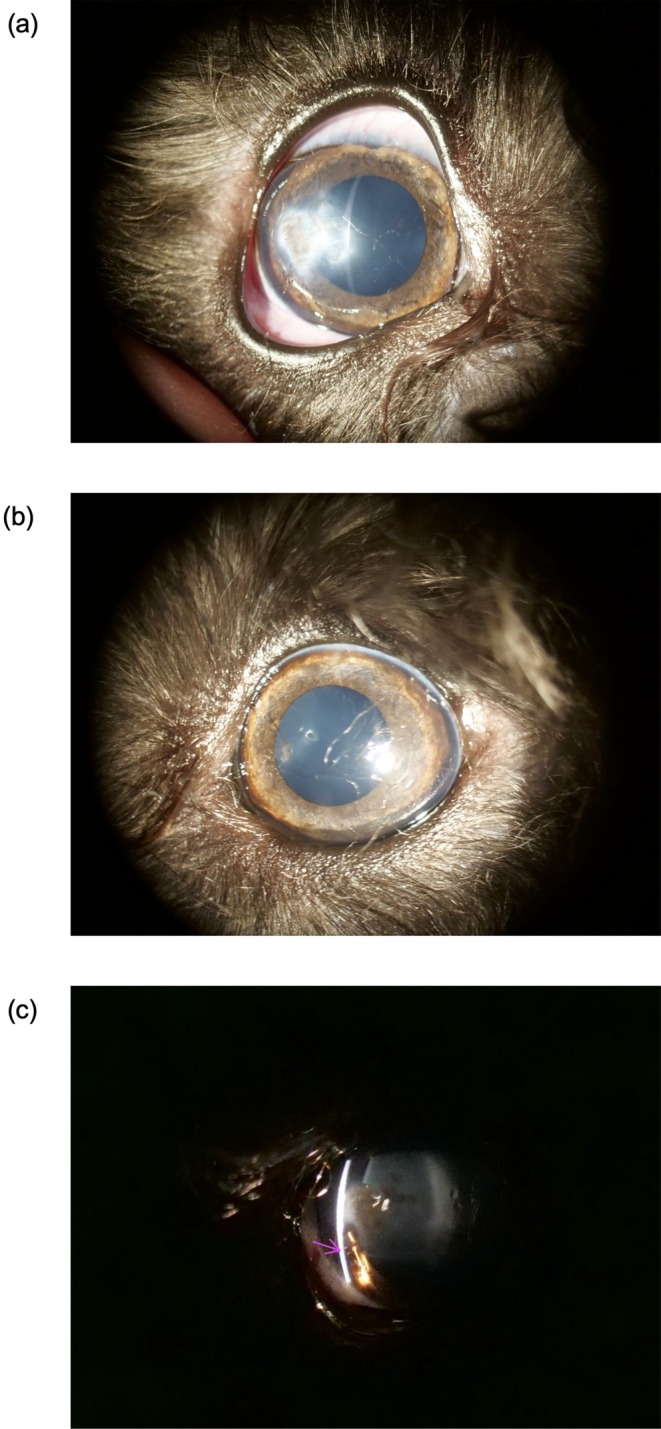
Slit‐lamp images of both eyes. (a) Right eye showing a broad paraxial corneal opacity. (b) Left eye with a clear cornea and no abnormal adhesion; the linear structure visible in the image represents a superficial hair artifact on the ocular surface at the time of imaging. (c) Magnified view of the right eye demonstrating iridocorneal adhesions extending from the iris collarette to the posterior cornea (arrows).

A 10‐month‐old intact male Miniature Schnauzer weighing 6.02 kg was presented for evaluation of a corneal opacity in the right eye (Figure 1). The breeder had relinquished the dog early in puppyhood due to the ocular appearance. The current owner reported no history of ocular pain, ocular discharge, or systemic illness. Vaccinations and parasite control were up to date. General physical examination was unremarkable.

## 3 Differential Diagnosis and Investigation

All ophthalmic examinations and imaging procedures were performed, with the dog manually restrained and without sedation. Slit‐lamp biomicroscopy was performed using a slit‐lamp biomicroscope (Haag‐Streit AG, Koeniz, Switzerland). Intraocular pressure was measured using a rebound tonometer (TONOVET‐Plus; Icare Finland, Helsinki, Finland). B‐scan ultrasonography was performed using a diagnostic ultrasound system (Aplio me V; Canon Medical Systems, Tochigi, Japan) with an 18‐MHz linear probe, and magnified anterior segment images were obtained using the zoom function of the B‐scan ultrasound system. SD‐OCT imaging of the anterior segment was performed using a commercial SD‐OCT system (Spectralis; Heidelberg Engineering, Heidelberg, Germany).

## 4 Outcome and Follow‐Up

### 4.1 Ophthalmic Examination

Neuro‐ophthalmic testing revealed normal findings in both eyes (OU), with positive menace response, positive dazzle reflex, and positive direct and consensual pupillary light reflexes. The Schirmer tear test values were 22 mm/min OD and 23 mm/min OS. The intraocular pressure, measured with a rebound tonometer, was 11 mmHg in OD and 14 mmHg in OS. Slit‐lamp biomicroscopy of OD revealed a broad paraxial corneal opacity, with several strands of tissue extending from the iris collarette to the posterior cornea (Figure 1). The anterior corneal stroma appeared clear, while the opacity was localized predominantly to the posterior stroma and Descemet's membrane region. The corneal epithelium was intact, with no vascularization. The anterior chamber was of normal depth, and the lens appeared clear. In contrast, OS was unremarkable. Fundus examination using a handheld fundus camera showed no abnormalities in OU.

### 4.2 B‐Scan Ultrasonography

**FIGURE 2:**
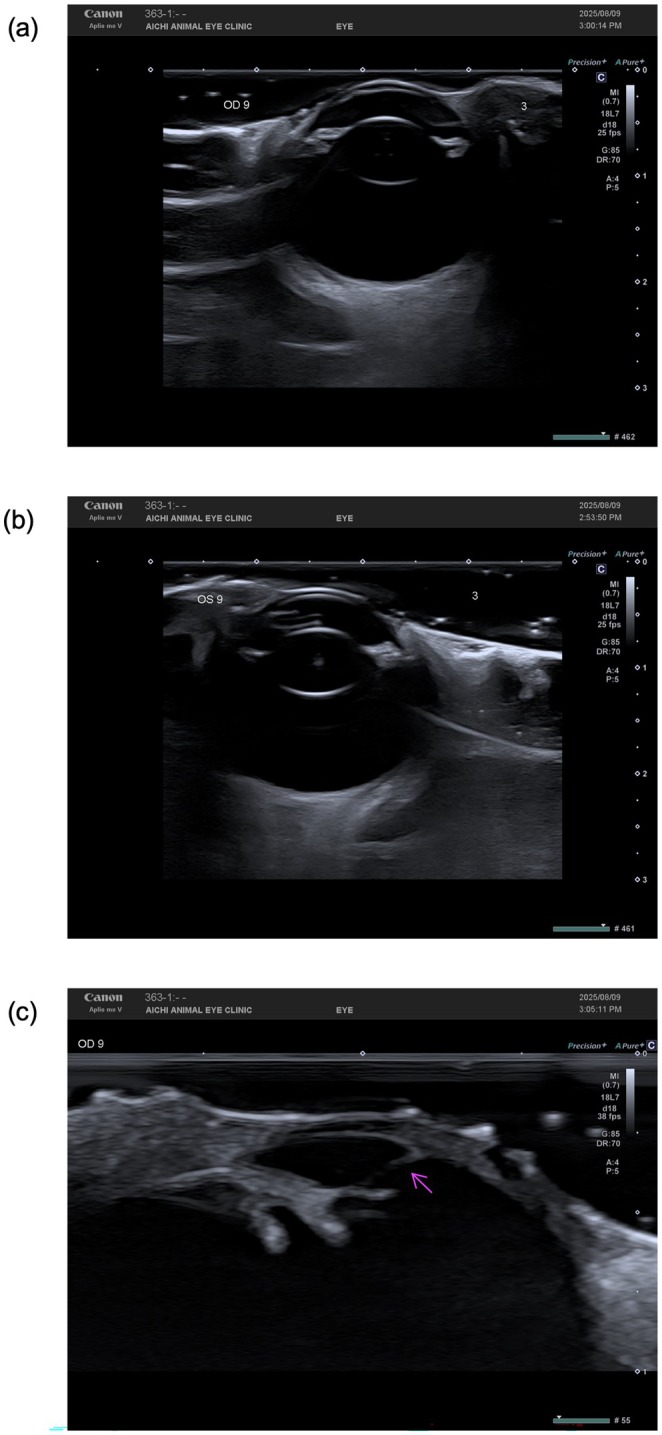
B‐scan ultrasonography of both eyes. (a) Right eye showing a normal lens and posterior segment. (b) Left eye showing a normal lens and posterior segment with no abnormalities. (c) Magnified view of the right eye anterior segment demonstrating several strands of tissue extending from the iris collarette to the posterior cornea (arrows).

B‐scan ultrasonography demonstrated normal posterior segment structures in both eyes (OU) (Figure 2). No evidence of lens abnormalities, vitreous opacity, or retinal detachment was detected. Magnified imaging of the anterior segment in OD revealed strands of tissue extending from the iris to the posterior cornea.

### 4.3 Spectral‐Domain Optical Coherence Tomography (SD‐OCT)

**FIGURE 3:**
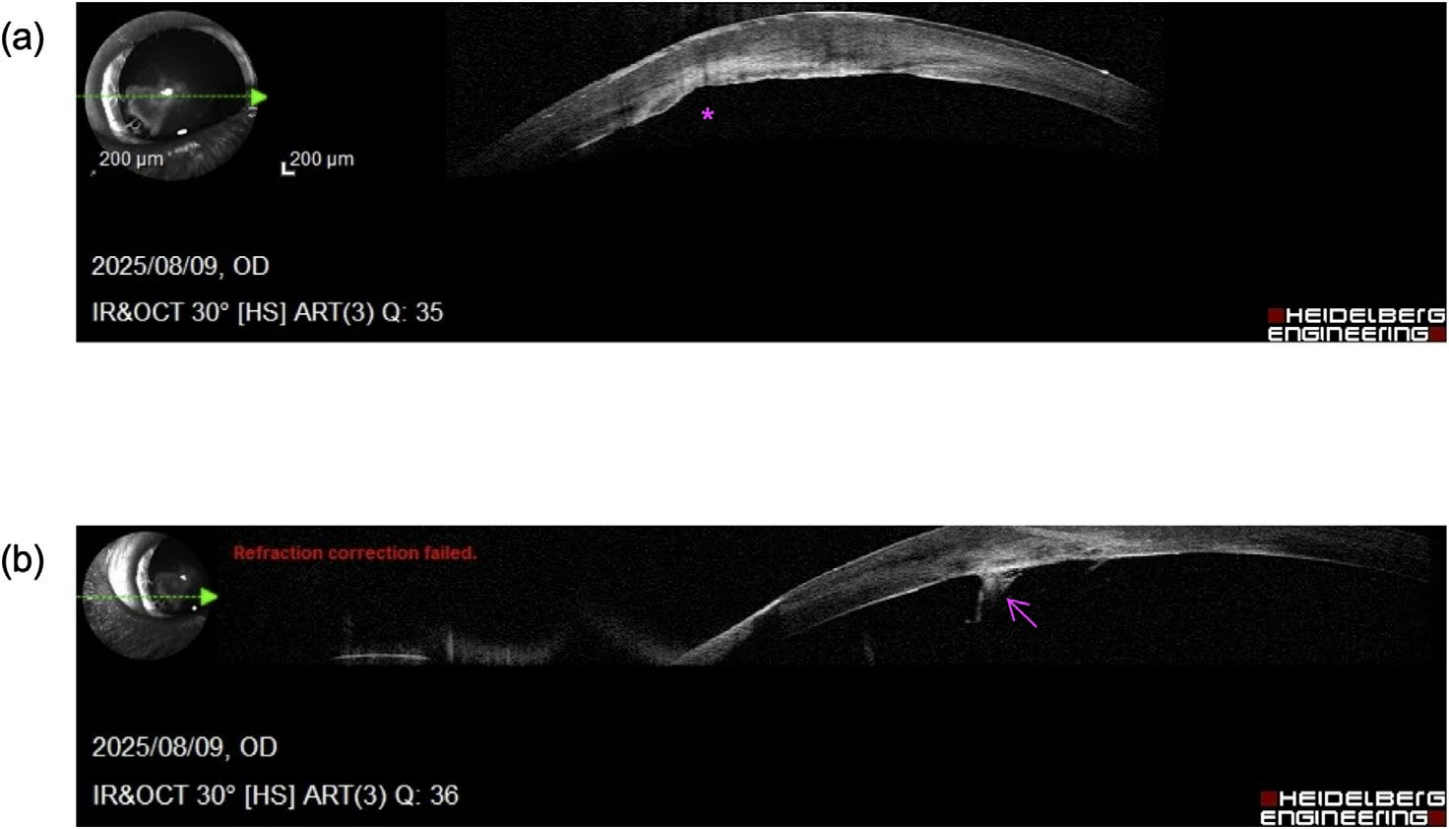
Spectral‐domain optical coherence tomography of the right eye. (a) Right eye showing focal absence of Descemet's membrane and corneal endothelium at the site of the opacity (asterisk), with an irregular posterior corneal contour. (b) Right eye demonstrating a strand of tissue attached to the posterior corneal surface (arrow).

SD‐OCT of OD revealed a focal absence of the Descemet's membrane and corneal endothelium corresponding to the area of corneal opacity. The posterior corneal surface in this region appeared irregular, and hyperreflective bands were observed extending from the iris collarette to the posterior cornea, consistent with iridocorneal adhesions (Figure 3). SD‐OCT of OS showed normal corneal layers and anterior segment anatomy.

Based on the clinical and imaging findings, we diagnosed this case as Peters anomaly.

## 5 Discussion

Peters anomaly is classified into two types in human medicine: type I, in which the lens is clear or only mildly opaque, and type II, in which the lens is cataractous and adherent to the cornea [2]. The present case is consistent with type I, given the absence of lens opacities and normal posterior segment findings. The embryologic basis involves abnormal migration or differentiation of neural crest‐derived mesenchymal cells, which form the corneal endothelium, Descemet's membrane, and trabecular meshwork [2]. This leads to localized absence of the Descemet's membrane and endothelium, as confirmed in our current case by SD‐OCT. The novelty of the present case lies in the in vivo visualization of focal Descemet's membrane and corneal endothelial defects using SD‐OCT, findings that have previously been described only in histopathological studies.

In addition to developmental anomalies during embryogenesis, genetic factors have been implicated in Peters anomaly pathogenesis. This rare congenital malformation of the anterior segment has been documented in humans, cats, and dogs [4, 5]. Several genes, including PAX6, PITX2, CYP1B1, FOXC1, COL6A3, SOX2, PITX3, and FOXE3, have been reported to be associated with the development of Peters anomaly in human patients [3]. Mutations in these genes are known to affect ocular morphogenesis, particularly in the migration and differentiation of neural crest cells, potentially leading to the structural defects observed in the cornea and anterior segment [1]. While genetic testing was not performed in our case, the possibility of a hereditary component cannot be excluded, and further molecular studies could help elucidate the underlying cause in canine patients.

In veterinary medicine, few cases of canine Peters anomaly have been described [5], and to the authors' knowledge, our study is the first case documented by SD‐OCT. OCT enabled high‐resolution, in vivo visualization of corneal layer absence and iridocorneal adhesions, consistent with histopathological descriptions in general reports [1]. The ultrasonographic detection of iridocorneal strands in the anterior segment further supports the diagnosis and demonstrates that non‐invasive imaging can yield complementary findings.

Although our current case was clinically stable without signs of discomfort, the long‐term prognosis remains uncertain, as secondary glaucoma or corneal decompensation may occur over time. Regular monitoring is warranted to detect progression or any complications.

## Author Contributions

**Yukihiro Miwa:** conceptualization, data curation, formal analysis, project administration, resources, supervision, writing – review and editing. **Deokho Lee:** data curation, formal analysis, visualization, writing – original draft, writing – review and editing. **Yuri Inukai:** data curation, formal analysis.

## Funding

The authors have nothing to report.

## Ethics Statement

This study was conducted in accordance with the code of ethics of the relevant Veterinary Medical Association.

## Consent

written informed consent was obtained from the owner to publish this report in accordance with the journal's patient consent policy prior to general ocular examination.

## Conflicts of Interest

The authors declare no conflicts of interest.

## Data Availability

The datasets generated and/or analyzed during the current study are available from the corresponding author on reasonable request.
